# Next Generation Reproductive and Developmental Toxicology: Crosstalk Into the Future

**DOI:** 10.3389/ftox.2021.652571

**Published:** 2021-03-18

**Authors:** Karin Sørig Hougaard

**Affiliations:** ^1^National Research Centre for the Working Environment, Copenhagen, Denmark; ^2^Department of Public Health, University of Copenhagen, Copenhagen, Denmark

**Keywords:** cumulated exposure, *in vitro*, placenta, pregnancy, fertility, future, developmental toxicology, reproductive toxicology

## Introduction

We have come a long way since the first case reports of lifelong male infertility following occupational exposure to dibromochloropropane, and the discovery of the debilitating developmental toxicity of thalidomide, methyl mercury, and alcohol (Potashnik and Porath, 1995; Kalter, 2003). The realization that chemicals can cross the placenta and inflict irreversible damage to the fetus and reproductive organs triggered concern for human health and made scientists and regulators strive for greater understanding of reproductive and developmental toxicity to protect future parents and children.

Today, more than 20% of couples will experience infertility (Mascarenhas et al., 2012). In Denmark, assisted reproduction helped in the conception of more than 10% of all children. Of all children, 6% are born too early, 5% with malformations, and 6% weigh too little at birth. In addition, children increasingly develop functional disorders such as asthma and attention deficit disorders (Sundhedsstyrelsen, 1997; Nielsen and Javid, 2014; Kommunernes Landsforening, 2019; Sundhedsdatastyrelsen, 2020). However, we lack knowledge about the extent of the effect of environmental chemicals in reproductive adversity. So much remains to be learned!

## Reproductive and Developmental Toxicity

Reproductive toxicity refers to effects on both fertility and development, but also refers to effects on fertility alone. Developmental toxicity refers to chemicals' interference with normal development of the organism, originating from exposure of either parent prior to conception, or exposure of the developing organism (ECHA, 2017). Early research focused on malformations and fetal death. Now it is broadly accepted that the gestational environment might also determine the developmental trajectories of the fetus and, hence, even health later in life, c.f. the concept of Developmental Origin of Health and Disease (Schmitz-Felten et al., 2016; Heindel et al., 2017). Parental exposure prior to birth has received less attention than gestational exposure, but the interest in e.g., male mediated developmental toxicity seems to have increased with the emergence of epigenetic programming, including sperm DNA and histone modifications and non-coding RNAs in spermatozoa (Bonde et al., 2019; Marcho et al., 2020).

## The Challenge(S)

The number of chemical substances falling under REACH (Registration, Evaluation, Authorisation, and Restriction of Chemical substances in Europe) ranges from 70,000 to 100,000, to which substances such as pharmaceutical compounds should be added. Testing for reproductive and developmental toxicity is governed by international guidelines, primarily from the Organization for Economic Co-operation and Development (OECD) and the International Council for Harmonization of Technical Requirements for Registration of Pharmaceuticals for Human Use (ICH) (Estevan et al., 2017; European Medicines Agency, 2017; OECD, 2018).

Experimental toxicology has the advantage of control over exposure, environment, and genetics, but high cost and extensive use of experimental animals preclude a complete assessment of reproductive and developmental toxicity for all chemicals (Rovida and Hartung, 2009). REACH has increased toxicity testing of industrial chemicals, but data on reproductive and developmental toxicity is still very limited, as only very high tonnage levels (>1,000 tons/year) triggers extensive testing (ECHA, 2017). In addition, not even the most extensive test protocols guarantee detection of all inherent developmental and reproductive toxicity. Some outcomes are too rare for detection of effect at the recommended group sizes, and assessment of offspring organ function is limited. The OECD Extended One-generation Reproductive Toxicity Study evaluates offspring fertility and some indicators of endocrine disruption and immune- and neurofunction (OECD, 2018), but not whether exposure elevates risks of the several highly prevalent human health problems that occur later in life, such as asthma, diabetes, cardiovascular disease, and dementia. Developmental neurotoxicity has a separate and extensive guideline, but it is rarely used (Makris et al., 2009). Also for pharmaceuticals, a very limited range of functional outcomes are assessed in the offspring (European Medicines Agency, 2017). In this respect, maternal airway exposure to nanomaterials presents an interesting case. Findings in several studies indicate that gestational and litter parameters might be less sensitive to maternal particle exposure than offspring organ function in postnatal life (Hougaard et al., 2015; Larsen et al., 2020). We ought to study whether this also applies to “traditional” chemicals.

Furthermore, test requirements relate mainly to compounds in production, and not to the myriad of compounds generated by anthropogenic processes such as fumes from combustion, welding, or e-cigarettes. Testing of such factors is largely in the hands of dedicated university and governmental researchers (Schmitz-Felten et al., 2016).

The emerging understanding that exposure from multiple sources can interact adds to the complexity. Pioneer work on endocrine disrupting chemicals made clear that chemicals, which individually exert no or small effects, might induce marked responses in concert (Hass et al., 2007). This was later confirmed in several studies (Martin et al., 2020). Also, non-chemical stressors might enhance developmental adversity of chemicals (Hougaard and Hansen, 2007; Sobolewski et al., 2018). The EU presently evaluates chemicals one by one, but now recognizes risks from multiple chemical exposures must be taken into account, e.g., in workers enduring chemicals in both private and occupational life (European Commission, 2020), not to forget pharmaceuticals. Overall, consumers and workers risk using chemicals without or with limited knowledge on toxicity to reproduction.

### The Alternative Road(s)

Acknowledging the need for both toxicological testing and reduced use of experimental animals, hazard prediction based on limited or even no testing in biological systems is urgently needed (Rovida and Hartung, 2009; Scialli et al., 2018; Clements et al., 2020). This is challenged by the complex reproductive cycle: gametogenesis, fertilization, implantation, and development until sexual maturity, involving the precise coordination of multiple processes in several organs, in parents and offspring, and even the generation of a new organ, the placenta (Parks Saldutti et al., 2013; Felter et al., 2015).

#### Refinement and Hypothesis Driven Testing

Rethinking reproductive and developmental toxicity testing takes several paths. One involves refinement of existing protocols, by increasing the accuracy of assessments and dose selection, testing of metabolites rather than mother compounds, read across, implementing critical windows of sensitivity, and humanization of animal models (e.g., by CRISPR technology) etc. (Scialli et al., 2018).

Another path applies hypothesis driven testing. Consensus is growing that understanding of toxicological mechanisms can feed into and accelerate development and applicability of alternative test methods. A logical step is identification of key events that predict adverse reproductive outcomes, e.g., based on structure-activity relationships, high throughput screening, and toxicogenomics (Scialli et al., 2018), to feed into adverse outcome pathways (AOPs), as recently described for female reproductive disorders (Draskau et al., 2020; Johansson et al., 2020) and the role of retinoic acid in embryo development (Tonk et al., 2015). AOPs sharing key events can combine into networks that ultimately capture all events on the path to specific outcomes. AOP networks are especially important in developmental toxicity that rarely arises as a single, linear chain of events, but rather by disturbances in the internal balance of a multitude of competing factors and interacting cell types changing with time and location (Tonk et al., 2015). When AOPs are established, chemicals can be tested *in vitro, in chemico*, or *in silico* relative to the crucial events rather than by standard (animal) protocols.

#### Non-animal Test Systems

Combining mechanisms of toxicity with knowledge of specific compounds and their close analogs can then form the basis for development and selection of targeted experimental approaches, such as composition of organotypic models, as testis-on-a-chip (Parks Saldutti et al., 2013; Baert et al., 2020), and batteries of complementary *in vitro* assays. Some batteries are directed toward one organ or outcome, e.g., developmental neurotoxicity (Bal-Price et al., 2018), while others are more generalized. One included the CALUX transcriptional activation assay (steroidogenic activity), ReProGlo assay (body axis patterning and cell fate specification), embryonic stem cell test (differentiation into cardiomyocytes), and zebrafish embryotoxicity assay. The battery predicted developmental toxicity correctly for most tested compounds and was useful for verification of read-across between structurally related chemicals (Kroese et al., 2015). The latter approach introduces hypothesis-driven testing, where knowledge about compounds or close analogs directs testing toward potential modes of action instead of the one-protocol-fits-all approach (Scialli et al., 2018).

Unfortunately, most model systems of developmental toxicity lack the placenta. Only recently has the placenta's many active roles become evident. Placental toxicology is still in its infancy, but accumulating data associates environmental exposures to microvascular dysfunction and adverse pregnancy outcomes (Gingrich et al., 2020). In animals, particles have been shown to perturb the placental environment, *via* induction of oxidative stress and inflammation (D'Errico and Stapleton, 2019; Dugershaw et al., 2020), and human exposure to (particulate) air pollution increases the risk of hypertensive disorders in pregnancy (Pedersen et al., 2014). The *ex vivo* human placenta model provides a mean for assessment of placental transfer in humans (Aengenheister et al., 2020), and advanced placental models envision recreation of the intrauterine architecture in its entirety (Pollet and den Toonder, 2020).

Induction of epigenetic changes constitutes a potential mechanism by which chemicals can influence reproduction and development. Epigenetics refers to stable changes in gene expression without modification of the DNA sequence. Chemical exposure may alter the epigenetic make-up, and such “memory markers” of environmental insults can persist even after exposure is terminated. Epigenetic changes might even transmit to subsequent generations. Chemically-induced epigenetic changes do not suffice for regulatory classification of chemicals. Recognition of the potential for epigenetic moderation by chemicals allows for deeper understanding of the developmental origin of disease. Linkage of epigenomic changes to corresponding phenotypes could furthermore provide a “handle” for epigenetics in hazard identification and incorporation of epigenetic evaluation into toxicity tests (Ideta-Otsuka et al., 2017; Heindel, 2019).

There is still a ways to go until whole organ models and test batteries supply data at level with or more predictive than conventional methods (Clements et al., 2020). (Quantitative) Structure Activity Relationships [(Q)SAR] offer another route for prediction of reproductive and developmental toxicity, but the complexity of reproductive biology poses a challenge in individual models. Possibly, integration of several models might reduce uncertainty and improve predictions (Marzo et al., 2016). Check, for example, developmental toxicity or endocrine endpoints at the Danish (Q)SAR database (qsar.food.dtu.dk).

#### Population Based Studies

The environmental causes of congenital malformations were in many instances discovered in humans by coincidence (Kalter, 2003), and the Developmental Origin of Health and Disease concept emerged from the study of human birth cohorts (Heindel et al., 2017). Several birth cohorts have systematically and prospectively collected information on maternal exposure, pregnancy, and birth, and followed-up the children on a regular basis (Vrijheid et al., 2012). Children in some cohorts have even reached reproductive age, allowing for the study of developmental exposures on their own reproductive capability (Brauner et al., 2019; Haervig et al., 2020), and soon of health in generations. The cohorts, furthermore, allows for the study of associations in one cohort and the subsequent (re)confirmation of findings in other cohorts, to test for robustness and generalizability of findings.

We are moving away from “one exposure, one disease” toward a multifactorial understanding of reproductive and developmental health, including timing of exposure during the life cycle. For fertility, postponement of parenthood toward the end of the reproductive years, and hence, age-related decrease in especially female fecundity, is suggested to largely account for the increase in assisted reproduction (Baird et al., 2005). At this time in life, women and men (and their reproductive organs) have however also experienced more years of occupational and environmental exposures. Smoking, some chemicals, and chemotherapy accelerate menopause, but otherwise knowledge is scarce (Iorio et al., 2014). Of note, fertility might be more sensitive to disruption if already compromised, e.g., due to advanced age (Hjollund et al., 1999; Minguez-Alarcon et al., 2017). This indicates a need to consider exposure during multiple and potentially interacting windows during the life cycle. Also relevant is interaction of exposures *in utero* and in adulthood. Hence, prenatal exposure could alter susceptibility to exposure later in life and predispose an individual for disease (“two-hit hypothesis”) (Heindel, 2019). Unexposed and exposed offspring could even present as similar phenotypes, until challenged by stressors (Hougaard et al., 2005; Hansen et al., 2020).

In the study of multiple concomitant exposures and stressors, human cohorts can supply variation in (epi)genetics, timing, and exposures. Such analyses require large populations, and merging of (birth) cohorts might be necessary. Prospective studies are pivotal for understanding the impact of environmental exposures, but study of the full lifespan is hampered by human longevity (NRC, 2017; Nakayama et al., 2019). Here, nationwide registries of health, income, job, and place of living (such as nationwide Scandinavian registries starting in the 1970's), could be combined with exposure matrices, biomonitoring, and exposome analysis of biobanked material to provide a basis for advanced “big data” analyses and to generate hypotheses for study in the aging birth cohorts.

## The Future Lies in Crosstalk

The grand challenge lies in the enormous number of chemicals and pharmaceuticals that precludes establishing adequate databases for reproductive and developmental toxicity for all. We urgently need hazard prediction and risk assessment based on limited or no testing in biological systems. This requires a deeper understanding of the pathological pathways underlying disruption of fertility and fetal programming, to feed into, e.g., AOP networks and *in silico* methods. A library of *in vitro* test systems ought to ultimately cover the full reproductive cycle (and placental processes) as basis for selection of combinations of experimental techniques for targeted test batteries.

This requires knowledge of the *in vivo* relevance of the sometimes relatively simple *in vitro* endpoints, and on how to extrapolate *in vitro* concentrations to *in vivo* kinetics. We must however also discuss animal studies as the gold standard for human hazard assessment, look into the consequence of strain differences in toxic response (Skovmand et al., 2020), and review how findings and hypotheses generated in animals and *in vitro* correlate with evidence in humans (Bonde et al., 2016).

Testing of the consequences of early exposure for health later in life needs to include a broader palette of organ systems, such as offspring kidney and cardiovascular and immune systems. Causal association to effect biomarkers, such as epigenetic changes, must be established. Our view of sensitive periods must also include the preconception period for women and men alike, and interaction between exposures, concomitant as well as during different stages of life. Focus should shift from the several well- or even over-studied chemicals to those without a database (Grandjean et al., 2011) to new types of chemicals, and combinations of chemicals and common factors in everyday life: non-chemical factors in the occupational setting, psychosocial stress, nutrition, age, etc.

The overall goals can only be achieved through continuous crosstalk between researchers and methodologies from different disciplines, such as *in vitro* and experimental animal toxicology and epidemiology; basic and molecular biology; chemistry, pharmacology, and medicine; exposure assessment, occupational and environmental science; statistics, *in silico* modeling and systems biology; risk assessment, regulation and prevention, to mention a few.

One thing is certain—there is room to explore reproductive and developmental toxicology into the next century. This Specialty Section in Developmental and Reproductive Toxicology encourages the investigation of the outlined challenges—and their solutions. It invites review articles, primary research, and method papers for a broad range of readers within the many fields required to keep the soil fertile for development of this important field of research.

## Author Contributions

The author confirms being the sole contributor of this work and has approved it for publication.

## Conflict of Interest

The author declares that the research was conducted in the absence of any commercial or financial relationships that could be construed as a potential conflict of interest.
